# Use of cone-beam computed tomography to evaluate root and canal morphology of mandibular first and second molars in Turkish individuals

**DOI:** 10.4317/medoral.18473

**Published:** 2013-03-25

**Authors:** Sezer Demirbuga, Ahmet E. Sekerci, Asiye N. Dinçer, Muhammed Cayabatmaz, Yahya O. Zorba

**Affiliations:** 1 DDS, Research Assistant, Department of Conservative Dentistry and Endodontics, Faculty of Dentistry, Erciyes University, Kayseri, Turkey; 2 DDS, Research Assistant, Department of Oral and Maxillofacial Radiology, Faculty of Dentistry, Erciyes University, Kayseri, Turkey; 3 DDS, PhD, Associated Professor and Chair, Conservative Dentistry and Endodontics, Faculty of Dentistry, Erciyes University, Kayseri, Turkey

## Abstract

Objective: The aim of this study was to investigate the root and canal morphology of mandibular first and second molars in a Turkish population by using cone beam computed tomography (CBCT). Study design: CBCT images of mandibular first (n = 823) and second molar (n = 925) teeth from 605 Turkish patients were analyzed. The root canal configurations were classified according to the method of Vertucci. Results: The majority of mandibular molars (95.8% of first molars, 85.4% of second molars) had two separate roots; however, three roots were identified in 2.06% of first molars and 3.45% of second molars. C-shaped canals occurred 0.85% of first molars and 4.1% of second molars. Three canals were found in 79.9% of first molars and 72.8% of second molars. Most distal roots had a simple type I configuration, whereas mesial roots had more complex canal systems, with more than one canal. The most common root morphology of first and second molars is the two rooted morphology with three canals. Both the mesial and distal roots showed wide variations in canal anatomy with type IV and type I canal configuration predominating in the mesial and distal roots, respectively. Conclusion: Vertucci type I and IV canal configurations were the most prevalent in the distal and mesial roots, respectively, of both the mandibular first and second permanent molar teeth.

** Key words:**Cone-beam CT, Turkish, mandibular molars, root and canal morphology.

## Introduction

The knowledge of root canal anatomy has a major influence on the success rate of endodontic treatment. Successful root canal therapy consists of thorough biomechanical instrumentation and chemical debridement, followed by hermetic obturation of the root canal system. However, the complexity of the root canal anatomy presents clinical challenges and difficulties that often jeopardize the primary goal of such therapy. Therefore, variations in the root canal systems and characteristic features in different races should be recognized before or during endodontic treatment (1).

The internal anatomy of dental roots and canals has been assessed by canal staining and tooth clearing (2), plastic resin injection (3), conventional radiographs (4), digital and contrast medium-enhanced radiographic techniques (5), sectioning (6), in vitro macroscopic examination (7), in vivo root canal therapy with magnification (8), scanning electron microscopy evaluation (9), computed tomography (CT) techniques (10), micro-CT (11), and cone beam CT (CBCT) (12). CBCT, one of these methods, potentially provides the clinician with the ability to observe an area in three different planes with a practical tool for noninvasive and 3-dimensional (3D) reconstruction imaging for use in endodontic applications and morphologic analyses. The combination of sagittal, coronal, and axial CBCT images eliminates the superimposition of anatomic structures (13). Root morphology can be visualized in three dimensions, as can the number of root canals and their convergence or divergence from each other (14). From this point, CBCT has been suggested to assist in identifying root canal systems.

When reviewing the literature regarding root and canal morphology using the PubMed Database (National Library of Medicine), there were reports on the root canal morphology of permanent teeth in Turkish population. However, no detailed data of the root and canal morphology of the mandibular first and second molars in Turkish individuals have been found using CBCT for evaluation. The aim of this report was to analyze the root and canal morphology of these teeth in a Turkish population by using CBCT.

## Material and Methods

We designed a retrospective study composed of CBCT (Newtom 5G, QR, Verona, Italy) images of 785 patients who presented to the Oral and Maxillofacial Radiology service at the Erciyes University, Dentistry Faculty between June 2011 and March 2012. All records were selected from a Turkish population from the Cappadocia region of Turkey. CBCT images had been taken because of the patients’ previous dentomaxillofacial problems. Teeth were selected according to the following criteria: (i) permanent molars with no periapical lesions; (ii) no root canals with open apices, resorption or calcification and (iii) the CBCT images of good quality. The teeth with root canal fillings, restorations, posts, or crowns were exluded from study. The final sample group included data from 605 patients (337 females and 268 males).

The CBCT images were analyzed with the inbuilt software (NNT) in a Dell Precision T5400 workstation (Dell, Round Rock, TX, USA), with a 32-inch Dell LCD screen with a resolution of 1280 x 1024 pixels in a darkroom. The contrast and brightness of the images were adjusted using the image processing tool in the software to ensure optimal visualisation. A dentomaxillofacial radiologist and three endodontists evaluated concurrently all the images to reach a consensus in the interpretation of the radiographic findings. The presence of a root canal and the types of canal configurations root were evaluated using the NNT toolbar by carefully rolling downward through the images from the floor of pulp chamber to the apex to find out the number of roots, the number of root canals, and the canal configuration at the axial tomographic slices. Selecting and moving the cursor on 1 image to change the center of view altered the reconstructed slices in 3 orthogonal planes. Tomography sections of 0.150 mm in the axial, coronal, and sagittal planes were created. Axial and cross-sectional images (coronal and sagittal image) were transmitted to a personal computer in the digital imaging and communications in medicine (DICOM) format and reconstructed into multiplanar reconstruction images using the DICOM viewer: ExaVision SX Ver.1.13 (Ziosoft, Inc,Tokyo, Japan). These views were used to examine the root canal system.

The teeth involved were investigated radiographically by CBCT for the following observations were evaluated: (i) the number of roots and their morphology; (ii) the number of canals per root; (iii) the canal configuration in each root using Vertucci’s classification (15); (iv) the frequency of additional roots and the frequency of C-shaped canals in the mandibular first and second molars using Fan’s classification (11).

To check for the diagnostic reproducibility of the interreliability of the investigators, 10% of the radiographs assigned by them were randomly examined each day for three consecutive days. Examination of results using the Wilcoxon matched pairs signed-rank test showed no statistically significant differences between the two observers (a dentomaxillofacial radiologist and an endodontist), indicating diagnostic reproducibility.

The total number of roots and root canals, the root canal configuration, bilateral and unilateral appearance, the incidence, and the correlations between left- and right-side occurrences and between males and females were analyzed. Statistically significant differences were evaluated using the chi-square test with SPSS 16.0 for Windows (SPSS, Chicago, IL). P value of < 0.05 was considered statistically significant.

## Results

**Figure 1:**
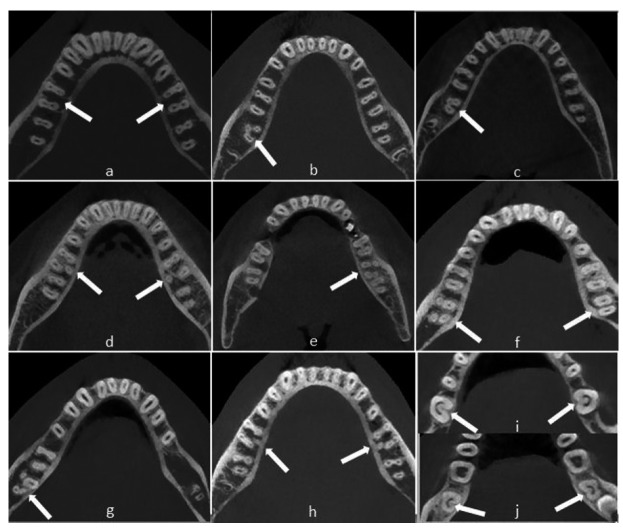
Cone-beam computed tomography images demonstrate: (a) Cases of mandibular bilateral first molars with two roots and two canals in axial section. (b) Case of mandibular right second molar with three root and mesiolingual root. Note that distal root fused with buccal part of mesial root (c) Case of mandibular right second molar with two roots. Note that distal root fused with buccal part of mesial root. (d) Distolingual roots can be seen concurrently in the first molars bilaterally. (e) Cases of mandibular left second molar with mesiobuccal 2-distobuccal 2 roots. (f) Cases of mandibular right second molar with four roots and left second molar with two roots- two canals. (g) Cases of mandibular right second molar with mesiobuccal 2-distolingual 2 roots. (h) Cases of bilateral mandibular first molars with five-canal and three roots. (i,j) Cases of bilateral mandibular first and second molars with C-shaped canals.

Of the patients enrolled, 337 females and 268 males, with a mean age of 35.7 years (SD:14.93), ranging from 15 to 78 years. For the mandibular first molar (823), 314 of the subjects had bilateral, 123 had unilateral molars, and 72 had no first molar. For the maxillary second molar (925), 376 of the subjects had bilateral, 173 had unilateral molars, and 46 had no second molar. The data for number of roots/canals and their morphology are presented in  Table 1, Table 2, Table 3. Variants in the root canal morphology of the mandibular first and second molars, without considering further the classification in each root were shown in figure 1.

**Table 1:**
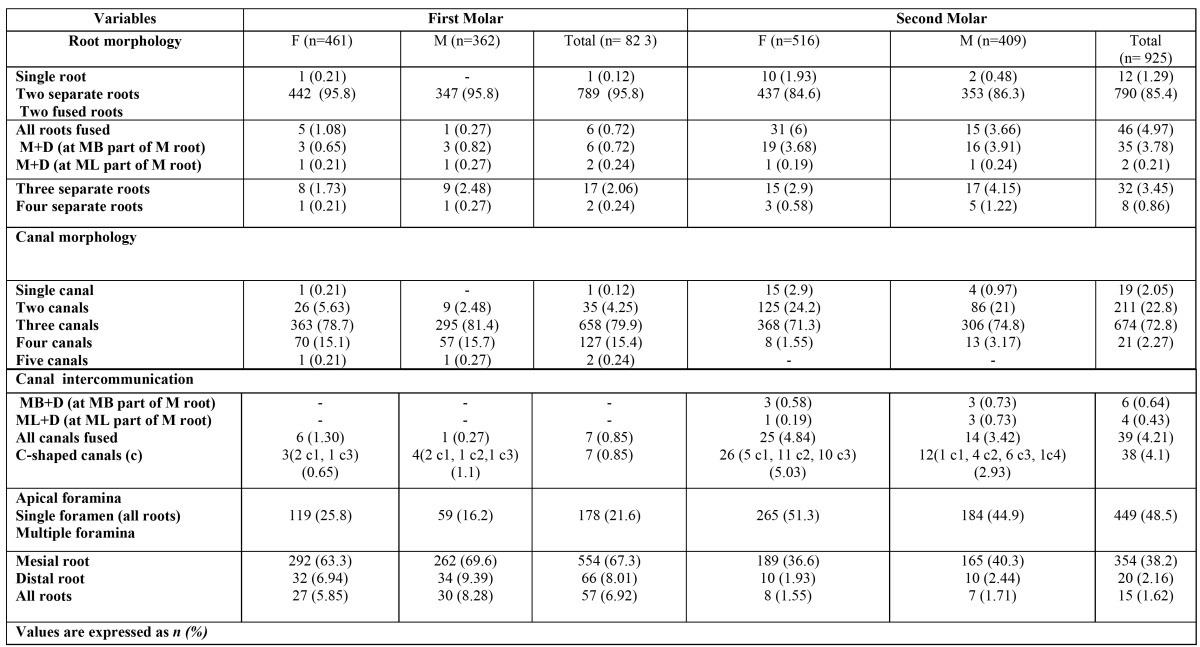
The frequency and distribution of root morphology and apical foramina of the root canals in mandibular first and second molar teeth.

**Table 2:**
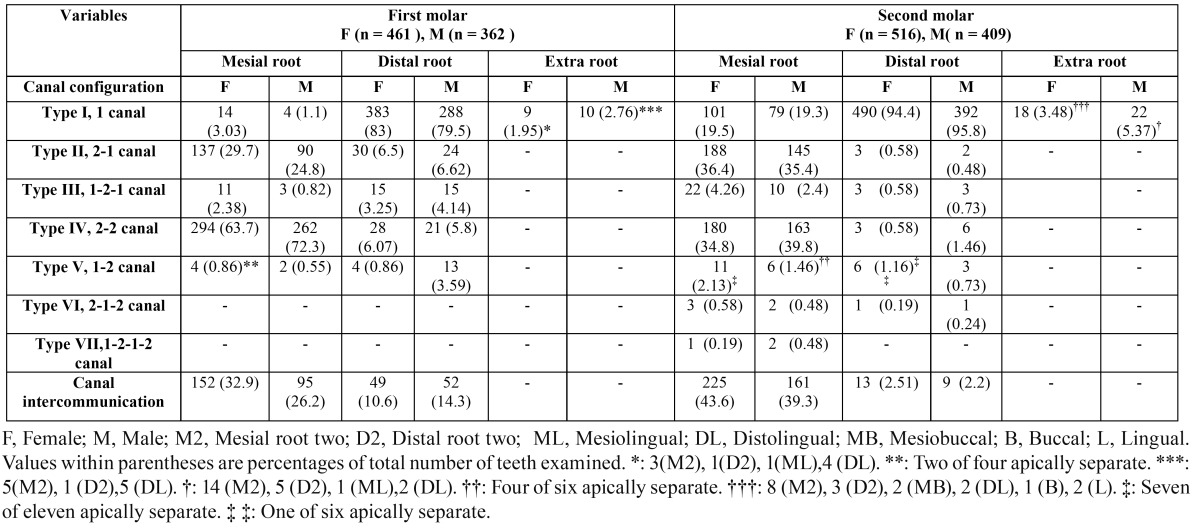
Frequency distribution of root canal configurations and canal intercommunications in mandibular first and second molar teeth.

**Table 3:**
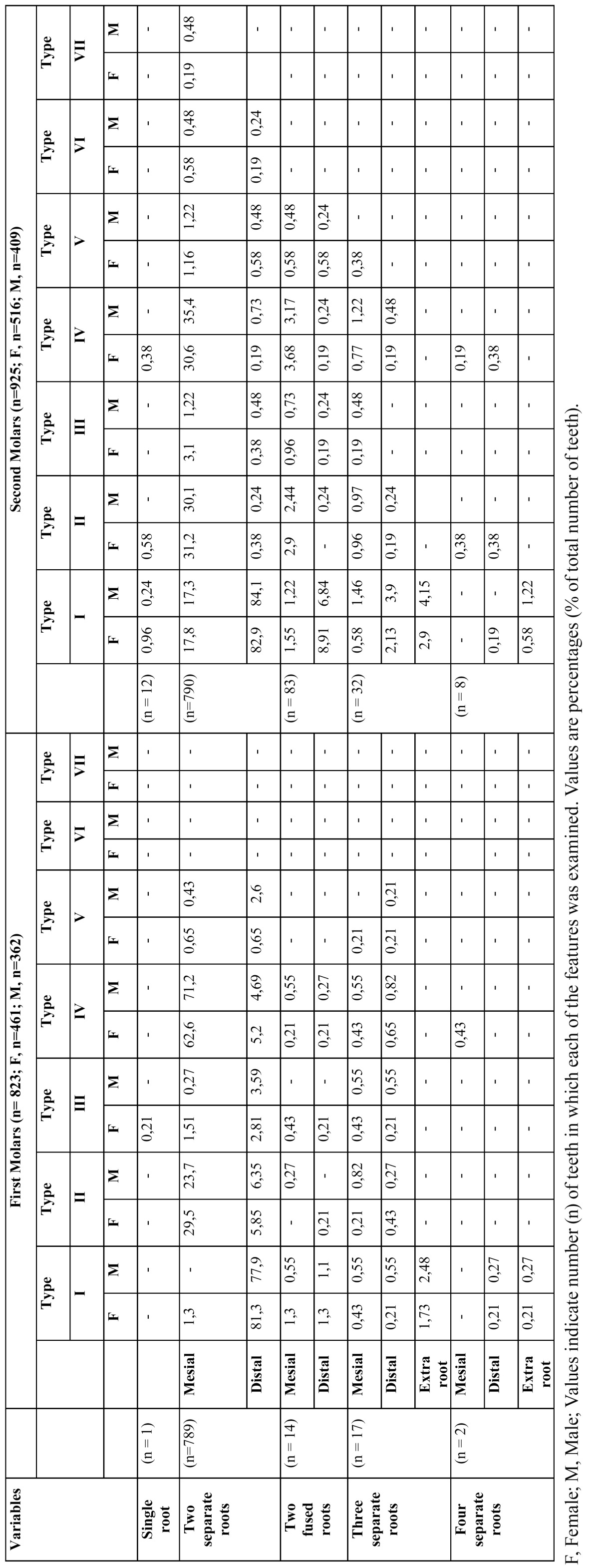
Number of Roots and Configuration of Root Canal Systems in Mandibular First and Second Molars.

-Mandibular first molars

The frequency distribution of roots according to sex is listed in  Table 1. The majority (95.8%) of mandibular first molars had two separate roots. The incidence of two separate rooted mandibular first molars was equal between in males and in females. Seventeen patients (2.06%) (8 females and 9 males) were detected to have a three-rooted mandibular first molar. When a third root was present, it was always on the lingual aspect of the main distal root. No statistical sex related difference (p >0.05) was detected for the incidence of three-rooted mandibular first molars. The incidence of one-rooted mandibular first molars was very rare (1/823, 0.12%) with only 2 four-rooted teeth were identified. Fused roots in teeth with two roots was found completely (0.72%) and partially (0.97%). Regardless of sex, the overall occurrence of the roots on the left side and the right side showed no statistically difference (p >0.05).

The incidences of root canals are listed in table 1. There were five variants in the root canal morphology of the mandibular first molars, without considering the further classification in each root. The distributions and percentages of the five categories of variants in the root canal anatomy of the mandibular first molars are listed in  Table 2. In total, 79.9% of the mandibular first molars had three canals, 15.4% had four canals, and 4.25 had two canals. The remainder had either five canals (0.24%) or single canal (0.12%) ( Table 1). The frequency distribution of the number of root canals did not differ between females and males (p >0.05). The correlation between left- and right-side occurrences revealed no difference either (p >0.05). C-shaped canals were found 0.65% (2 c1 type, 1 c3 type) in females and 1.1 % in males (2 c1 type, 1 c2 type and 1 c3 type). A single apical canal foramen was more frequent in females when compared with the males: 25.8% vs. 16.2%. (p< 0.05) Multiple apical canal foramina (deltas) were more frequently found in males than females. Multiple apical canal foramina were not observed in the additional roots ( Table 1).

The results of the evaluations of the root canal systems are shown in  Table 2 and  Table 3. A total of 447 teeth (96.9%) had two mesial canals and demonstrated a wide variation of canal configurations. The most common were type IV (63.7% in female, 72.3% in male) and type II (29.7% in female, 24.8% in male). Of the distal roots, 83% had one canal and 16.7% had two canals of which type II (6.5%) and type IV (6.07%) were most prevalent in females, and 79.5% had one canal and 20.2% had two canals of which type II (6.62%) and type IV (5.8%) were most prevalent in males. There were no teeth with three mesial or distal canals. In the three or four rooted molars, all disto-lingual roots (100%) possessed type I canal configuration. Overall, 2.3% of these teeth had additional (distolingual or mesiolingual) roots. All of these roots had the type I configuration. The incidences of varying root canal configurations did not differ between females and males or between left- and right-side occurrences. The highest incidence of intercanal communications was found in the mesial roots (30%) followed by distal roots (12.2%) of two-rooted molars.

-Mandibular second molars

The frequency distribution of roots according to sex is listed in  Table 1. Most of the mandibular second molars (85.4%) had two separate roots, whereas 1.29% had one root. Thirty-two patients (3.45%) were detected to have a three-rooted and only eight molars had four roots. When a third root was present, it was always on the lingual aspect of the main distal root. No statistical sex related difference (p >0.05) was detected. Fused roots in teeth with two roots was found completely (4.97%) and partially (0.97%). The overall occurrence of the fused roots on the first and second molar showed statistically difference (p < 0.05).

The incidences of root canals are listed in  Table 1. There were four variants in the root canal morphology without considering the further classification in each root. The distributions and percentages of the four categories of variants in the root canal anatomy are listed in  Table 1. In total, 72.8% of the mandibular second molars had three canals, 22.8% had two canals, and no had five canals. The frequency distribution of the number of root canals did not differ between females and males (p >0.05). The correla-tion between left- and right-side occurrences revealed no difference either (p >0.05). C-shaped canals were found 5.03% (5 c1 type, 11 c2, 10 c3 type) in females and 2.93 % in males (1 c1 type, 4 c2 type, 6 c3 and 1 c4 type). A single apical canal foramen was more frequent (p< 0.05) in females when compared with the males: 51.3% vs. 44.9%. Multiple apical canal foramina (deltas) were more frequently found in males than females. Multiple apical canal foramina were not observed in the additional roots ( Table 1).

The results of the evaluations of the root canal systems are shown in  Table 2 and  Table 3. In females and males, a total of 447 teeth (96.9%) and 357 (98.6%) had two mesial canals, respectively and demonstrated a wide variation of canal configurations. The most common were type IV (34.8% in female, 39.8% in male) and type II (36.4% in female, 35.4% in male). Of the distal roots, 94.4% (in females) and 95.8% (in males) had one canal. There were no teeth with three mesial or distal canals. In the three or four rooted molars, all disto-lingual roots (100%) possessed type I canal configuration. Overall, 4.32% of mandibular second molar teeth had additional (distolingual or mesiolingual) roots. All of these roots had the type I configuration. The incidences of varying root canal configurations did not differ between females and males or between left- and right-side occurrences.

## Discussion

This study provides a detailed report on the root canal morphology of mandibular first and second molars in a Turkish population by using CBCT. The CBCT provides comprehensive information about the root canal from different directions that could not be detected using conventional radiographs or clinical techniques. Prior knowledge of root and canal anatomy facilitates the accurate detection of all root canals in a tooth during endodontic treatment. Although variety techniques have been used in studies evaluating canal morphology, the recent use of CBCT has made it possible to conduct a nondestructive 3D global analysis of the external and internal morphology of the root and canal system. According to present study, the sum of roots and root canals can be visualized clearly in axial sections. The multiplaner CBCT scans obtaining from axial sections could be a useful tool for the study of the anatomy of root canals without surgical intervention.

It is now generally accepted that the most common form of mandibular first molar has two roots located mesially and distally and three canals: two of them being located in the mesial root and one root canal in the distal root (16), but in populations with mongoloid traits, an additional root located distolingually is considered to be a normal morphologic variant and can be identified as an Asian trait (17). Gulabivala et al. (2) found a lower prevalence of two-rooted mandibular first and second molar teeth in a Burmese population (89.9% and 58.2%, respectively). Sperber and Moreau (9) observed a third root in 3.1% of 480 African Senegalese mandibular first molar teeth.

According to Skidmore and Bjorndal (3), one third of mandibular first molars have four canals. The variation in four canals mandibular first molars was reported to be from 26.0% to 57.7% by Fabra-Campos (18), also reported a great variance in the number of canals in the distal root. They found that a percent up to 47.6% of the distal roots have two canals. Martínez-Berná and Badanelli (19) found four canals in this root. The second distal root canal is an anatomic anomaly, despite being most frequently encountered (19). Vertucci and Williams (15) reported the occasion of two canals of the mesial or distal canal in the mandibular first molar merging into one, which joined at 10% of the middle third of the root, 60% merged in the apical third and 30% came together at the apical foramen. In the present study, most commonly observed root morphology was the two separate rooted mandibular second molars (85.4%), which is higher than the prevalence in the Burmese (58.2%) and Thai (54%) populations (20). These two-rooted second molars had one distal canal and two mesial canals, which either had a separate course or combined in the middle third of the root or apically. In the present study, the frequency of two canals in the mesial and distal roots of the first molar tooth was 97.8% and 17.8%, respectively, whereas the second molar tooth had two canals in 80.2% and 3.39% of the mesial and distal roots, respectively.

Mandibular first molars with three roots were observed in 2.06% of the teeth examined ( Table 1). This incidence of three-rooted mandibular first molars was very lower than that reported in previous studies of Japanese (22.7%) (21), Hong Kong (15.0%) (18). This variant has a frequency of less than 5% in white people (British, Dutch, German, Finnish and other European), African (Bushmen, Bantu,Senegalese), Eurasian and Indian populations (2). The presence of a third root is a typical variation in Chinese mandibular first molars. The high prevalence (29%) of third roots was reported by Zhang et al. (22). In present study, mandibular second molars with three roots were observed in 3.45% of the teeth examined. This data is lower than the prevalence in the Indian population (8.98%) (23) and higher than the report of Gulabivala et al. (20) in which no mandibular second molars with three roots were observed. The position of this third root was lingual which was agreement with the findings of Gulabivala et al. (2), who identified the distolingual root in first molars and proposed that this should be considered as a genetic trait and not a developmental anomaly. However, there is limited information on the root morphology related to the mandibular first molar with and without a distolingual root. Recently, it was reported that a high percentage of molar (21,5%) had a disto-lingual root in a dental radiographic survey in Taiwanese population (24). In the present study, the prevalence of distolingual root in mandibular first and second molar teeth was 1.09 % and 0.43%, respectively.

In the present study, the prevalence of root fusion in mandibular first and second molar teeth was 1.7 % and 8.97%, respectively. However, root fusion has been recorded in 19.1% of Thai mandibular first molar teeth (2) and in 52% of South Chinese mandibular second molar teeth (18).

In view of the significant differences among the results of studies on canal configuration and the prevalence of C-shaped canals in mandibular second molars in the different parts of the world differs (2.7-45.5%). C-shaped canals were first documented in the endodontic literature by Cooke and Cox (25) in three case reports. In this study, it was resulted that 7 out of 823 mandibular first molars (0.85%) and 38 out of 925 mandibular second molars (4.1%) had C-shaped canals. Future studies may reveal interesting information about the canal configuration and prevalence of C-shaped canals in mandibular second molars in different ethnic populations.

In the present study, multiple apical foramina were recorded in 75.3% (554 mesial, 66 distal) and 44.4% (354 mesial, 20 distal) of the first and second molar teeth, respectively (Table 1). These values are high compared with those in a Burmese population of 35.6% and 29.7% (20), of the first and second molar teeth, respectively. In the present study, intercanal communications (type II to type VII except type IV) were recorded in (348/823) 42.2 % and (409/925) 44.2% of the first and second molar teeth, respectively (Table 2). This figure is higher than the studies of Gulabivala et al. (20) (14.7% and 7.6%).

The root canal configurations of mandibular first permanent molars are diverse. In mesial roots, type IV configuration was most prevalent (63.7%) in females and (72.3%) in males followed by type II (29.7% in females and 24.8% in males). This is consistent with the findings of most of the earlier studies (26), except the studies by Zaatar et al. (27) and al-Nazhan (28) which reported type II being the most prevalent followed by type IV. Identification, preparation, and obturation of type IV, type II, and type VI are relatively straightforward. However, identification of canals in type V, where the canal further divides within the root, is more difficult. The presence of Gulabivala’s type (2-1-2-1) needs extra efforts, because failure to debride and disinfect this complex anatomy might have a direct effect on the treatment outcome. There are published reports indicating the presence of type VIII configuration in the mesial root, with the incidence of 0.2% to 5% (26). But in the present study, none of the samples had three canals in the mesial root. Gu et al. (29) examined 20 extracted three-rooted mandibular first molars in a micro-computed tomography study of Chinese patients. They found that almost all of the distolingual and distobuccal roots contained a type I canal. The results of the present investigation were in agreement with these studies.

The most common root canal configuration of second molar teeth in the present study was type IV (two canals, two foramina) in the mesial roots and type I (one canal, one foramen) in the distal roots (Table 2). This finding corroborates that of Ahmed et al. (30), but contrasts with those of Gulabivala et al. (20) and Vertucci and Williams (15). Pineda and Kuttler (4) recorded type I as the most frequent canal configuration in the mesial root of the second molar tooth in a Caucasian population. However, the most prevalent configuration of distal roots in the mandibular second molars was type I (95.3%) in present study. This finding corrobo-rates that of Ahmed et al. (30), Pineda and Kuttler (4), Gulabivala et al. (2) and Vertucci (15).

In conclusion, Vertucci type I and IV canal configurations were the most prevalent in the distal and mesial roots, respectively, of both the mandibular first and second permanent molar teeth.
